# Investigation of Citric Acid By-Products from Rice Produced by Microbial Fermentation on Growth Performance and Villi Histology of Thai Broiler Chicken (KKU 1)

**DOI:** 10.3390/vetsci8110284

**Published:** 2021-11-20

**Authors:** Mutyarsih Oryza.S, Sawitree Wongtangtintharn, Bundit Tengjaroenkul, Anusorn Cherdthong, Sirisak Tanpong, Padsakorn Pootthachaya, Wuttigrai Boonkum, Nisakon Pintaphrom

**Affiliations:** 1 Department of Animal Science, Faculty of Agriculture, Khon Kaen University, Khon Kaen 40002, Thailand; mutyarsih.or@kkumail.com (M.O.); anusornc@kku.ac.th (A.C.); sirisakt@kkumail.com (S.T.); padsakornp@kkumail.com (P.P.); wuttbo@kku.ac.th (W.B.); nisakon_m@kkumail.com (N.P.); 2 Department of Veterinary Public Health, Faculty of Veterinary Medicine, Khon Kaen University, Khon Kaen 40002, Thailand; btengjar@kku.ac.th

**Keywords:** citric acid rice, Thai broiler chicken, villi histology, microbial contamination

## Abstract

This study was conducted with Thai broiler chicken (KKU 1) to investigate the effect of citric acid by-products from rice (CABR) on growth performance and villi histology. A total of 192 broiler chicks were subject to three dietary treatments, including 0% CABR, 3% and 6% of dry matter. Body weight gains, feed intake, feed conversion ratio, survival rate, and production index (body weight gain, feed intake, feed conversion ratio, survival rates, and productive index, respectively) were considered for growth performance evaluation. Villi height (µm), crypt depth (µm), and villi: crypt ratio were recorded for the villi histological measurement. The performance did not show a significant effect when compared with the control group during at ages ranging from 1 to 56 days. Villi histology indicate a significant effect on villi height (µm), crypt depth (µm), and villi: crypt of broiler chicks compared with the control group. Also, the use of 3% CABR caused a reduction microbial contamination in chicken fecal matter. In conclusion, supplementation of CABR had no negative effects on growth performance of Thai broiler chicken (KKU 1). Also, the addition of 3% CABR to the feed might help reduce fecal microbial contamination and affect the villi histology of Thai broiler chickens (KKU 1).

## 1. Introduction

Citric acid is the world’s second-largest fermentation product and is both a weak and an essential organic acid. Commonly used as a feed additive, citric acid produces more than 1.7 million tons per year and is expected to increase every year [1]. Also, the demand for citric acid production has rapidly increased. Thus, the researcher and industries look for the raw materials that could reduce citric acid productions cost. Some study conducted for use some agriculture production to become citric acid as a raw material [2]. Nowadays in Thailand, rice is the major locally produced crop, which amounted to 20.7 million metric tons in 2019 [3]. Thus, rice certainly has the opportunity to be an ingredient to produce citric acid. The ability of rice and potato as an extract to produce citric acid was analyzed by Kudzai et al. [2] in order to meet the high demand for citric acid. The result was shown that rice could provide the highest production of citric acid, and rice extract media proved to be more useful than potato extract media. In China, rice has also become a popular raw material for citric acid production, and the obtained method was to use fermentation process on rice to produce citric acid [4]. Submerged fermentation with *Aspergillus niger* is commonly used to produce citric acid in the commercial sector [5]. The advantages of using *Aspergillus niger* over other potential citric acid producing microorganisms include its high citric acid productivity at low pH without toxic metabolite secretion, ease of handling, and ability to ferment a wide range of inexpensive raw materials. The ability of this fungus to convert existing sugars in the fermentation media to the citric acid is of very high efficiency, ranging from 70–90%. [6]. Obtaining citric acid production by the fermentation of rice using *Aspergillus niger* was studied by Kudzai et al. [2]. The result showed that rice extract media contains, and readily makes, more sugars available, resulting in higher citric acid production levels.

Besides, global citric acid production generates waste residue of the amount of 50–60% [7]. The waste from citric acid by-products (CABR) can cause pollution and environmental problems when not managed properly. The waste from citric acid by-products contains sugars, cellulose, hemicellulose, starch, and also protein, which can be utilized for animal feed. By-products from the citrus industry can make an important addition to the amount of locally produced feed for animals. The benefits of the by-product are directly related to its low-cost feed additive, which can lower the cost of animal feed when used as a feed substitute [8]. Citric acid by-products retain organic acids, which could be good for gut health and stimulate poultry immune responses [9]. Tanpong et al. [10] reported citric acid by-product from cassava could be an alternative energy source for animal feed, due to its containing 3.59 Mcal/kg and 6.11% of crude protein, which makes this by-product a possible alternative feed ingredient for animals and the advantage of this by-product is related to reduced feed cost, ecofeed, and reduction in waste from various industries. Mehdikhany et al. [11] reported that citric acid by-product contains 14.4% of crude protein and 4499.7 Kcal/kg of gross energy. This implies that the level of 5% citric acid by-product is suitable for broiler diets. S. Oryza et al. [12] reported that citric acid by-product from rice (CABR) contains 19.80% of crude protein, 11.97% of crude fiber, and 4005.72 Kcal/kg of energy, which implies that CABR could be utilized as an energy source for animal feed. Also, the result showed that CABR is not contaminated with aflatoxin or fumonisin, which makes this by-product possibly safe for animal feed. The objective of the present study was to utilize the CABR as a feed ingredient in Thai broiler chicken (KKU 1) in terms of growth performance and digestive performance, such as villi histology and microbial contamination on fecal matter.

## 2. Materials and Methods

### 2.1. Production Techniques of Citric Acid

CABR was obtained from the factory in the eastern part of Thailand and provided by the PS Nutrition Company Limited, Bangkok, Thailand. In the production, rice was used as a raw material substrate to produce citric acid by fermentation with A.nigger [13]. The fermentation process was of 144 h duration, under optimized temperature and moisture. First, rice as a substrate was milled and boiled before the fermentation process. The second step was adding the microbial (A.nigger) in the fermentation tank. After that, filtration process was used to separate the liquid and the waste product. The liquid was used to produce citric acid, and the waste product was dried in the sun or in an oven at the temperature of 95 °C. After the drying process, the CABR sample was used in this study.

### 2.2. Animals and Experimental Design

CABR was provided by the PS Nutrition Company Limited, Bangkok, Thailand. The chemical composition was here investigated in this study based on results from preliminary experiments, and then calculated for animal experiments [12]. The crude protein (CP) was 19.80% and gross energy (GE) was 4005.72 Kcal/kg.

This research used Thai broiler chicken (KKU 1) as an animal trial. A KKU 1 chicken is an indigenous hybrid chicken breed, developed by the Network Center for Animal Breeding and Omics Research. Thai broiler chickens have a terminal hybrid of 75% commercial broiler breed and 25% of Thai native chicken breed [14].

A total of 192 Thai broiler chicken (KKU 1) birds (one day old) and of mixed sex (male to female 1:1) were used. All animals used were consistent, and there were no differences in body weight throughout this study. The chickens were obtained from the Network Center for Animal Breeding and Omics Research, Faculty of Agriculture, Khon Kaen University, Thailand. The birds were randomly distributed into one of the three dietary treatments: (1) control group 0% of CABR, (2) 3% of CABR, and (3) 6% of CABR as corn replacement (Table 1) in a completely randomized design with four replications and 16 birds per pen or replication. This experiment was conducted on the Poultry Farm of the Faculty of Agriculture, Khon Kaen University, Thailand. This experiment provided water and feed ad libitum to birds for 56 days, divided into three of feeding periods, period: (1) period 1 (1–21 days), (2) period 2 (22–49 days), and period 3 (50–56 days).

### 2.3. Data Collection

#### 2.3.1. Growth Performance

During the study, the data such as body weight, body weight gain, feed intake, feed conversion ratio, and productive index (BW, BWG, FI, FCR, and PI, respectively) were recorded for each period and calculated for growth performance following the method of Singh [15]. All birds from each treatment were weighed weekly using electronic digital weighing machine to obtain the body weight. The amount of added feed to each pen and feed residue was recorded daily using electronic digital weighing machine. Feed consumption was calculated on a per period basis: (1) starter period (1–21 days), (2) growth period (22–49 days), (3) finishing period (50–56 days), and (4) overall period (1–56 days). BWG, FI, and FCR each period were calculated. In all trials, mortality was recorded and reported as a cumulative percentage and for PI, it was calculated following this formulation:

$$\;\mathrm{PI}=\frac{\mathrm{body}\;\mathrm{weight}\times \mathrm{survival}\;\mathrm{rate}\left(\%\right)\times 100}{\mathrm{age}\left(\mathrm{days}\right)\times \mathrm{FCR}}$$

Productive growth performance:

$$\mathrm{BWG}=\frac{\mathrm{final}\;\mathrm{weigh}\times \mathrm{initial}\;\mathrm{weight}}{\mathrm{number}\;\mathrm{of}\;\mathrm{birds}\;}\;\mathrm{FI}=\frac{\mathrm{Total}\;\mathrm{feed}\;\mathrm{consumption}}{\mathrm{number}\;\mathrm{of}\;\mathrm{birds}\;}\;\mathrm{FCR}=\frac{\;\mathrm{feed}\;\mathrm{intake}\;}{\mathrm{body}\;\mathrm{weight}\;\mathrm{gain}\;}\;\mathrm{Survival}\;\mathrm{rates}\;\left(\%\right)=\frac{\;\mathrm{Number}\;\mathrm{of}\;\mathrm{initial}\;\mathrm{birds}-\mathrm{Number}\;\mathrm{of}\;\mathrm{dead}\;\mathrm{birds}}{\mathrm{body}\;\mathrm{number}\;\mathrm{of}\;\mathrm{initial}\;\mathrm{birds}\;}$$

#### 2.3.2. Villi Histology

Following the method of De Verdal et al. [16], two birds of each treatment and each replication were sacrificed by cervical dislocation at the end of the experiment; sections from the middle of the duodenum, jejunum, and ileum were excised. The samples were fixed in 4% buffered formalin. The tissue processing consisted of dehydration, and clearing, with paraffin wax. Tissue sections, 5 μm thick (three cross-sections from each sample), were cut by a microtome and were fixed on slides. The sample was measured with an optical microscope (Eclipse E600, Nikon Corp., Tokyo, Japan) at 4× magnification. The microscope was fitted with a video camera (XC77E, Sony Corp., Tokyo, Japan) and the images were analyzed using image-analysis software Axio vision imaging system, 2018 version (Carl Zeiss, Co., Ltd., Seoul, Korea). The height of each villus was measured from the top of the villus to the crypt transition, and the crypt depth was defined as the invagination between two villi. The heights of 6 villi and the depths of 6 crypts were measured per animal (12 position of villus height and crypt depth per treatment).

#### 2.3.3. Microbial Contamination on Fecal Matter

In each of three replication trails, excrement samples were taken from the chicken cages at the end of the current experiment (56-day). The fecal content was pooled then examined at the Veterinary Diagnostic Laboratory Faculty of Veterinary Medicine Khon Kaen University, Thailand. A coliform/escherichia coli test was performed using the 3M Petrifilm Rapid *E.coli*/coliform counting plate, which was validated according to the AOAC Validation Guidelines following the AOAC Official Method of Analysis SM process [17]. A fecal sample was diluted with sterile saline peptone water in ratios ranging from 10:1 to 10:10, and the solutions were mixed thoroughly with a vortex mixer. One milliliter from each sample dilution was plated onto a single 3M Petrifilm Rapid *E. coli*/Coliform Count Plate. The plates were incubated at 37 °C for 18 to 24 h. After incubation, colony-forming units (CFU) were counted and recorded. The total coliform was count was indicated by red colonies as a result of gas production, while *E. coli* presented in blue color colonies both with and without gas production.

### 2.4. Statistical Analysis

The data were analyzed with a one-way analysis of variance (ANOVA) using the general linear model (GLM) based on SAS (SAS, Institute Inc., Cary, NC, USA, 2015) [18]. A completely randomized design was used for all parameters. Differences among means by Duncan’s new multiple range tests, with *p* < 0.05 were accepted as statistically significant differences.

## 3. Results

### 3.1. Growth Performance

The growth performance, initial weight, BWG, FI, FCR, SR, and production index (PI) of Thai native chicken fed with CABR was performed in Table 2. The results reveal that supplementation with CABR at 3 to 6% in the diet had no effect on growth performance (*p* < 0.05).

### 3.2. Villi Histology

**Figure 1:**
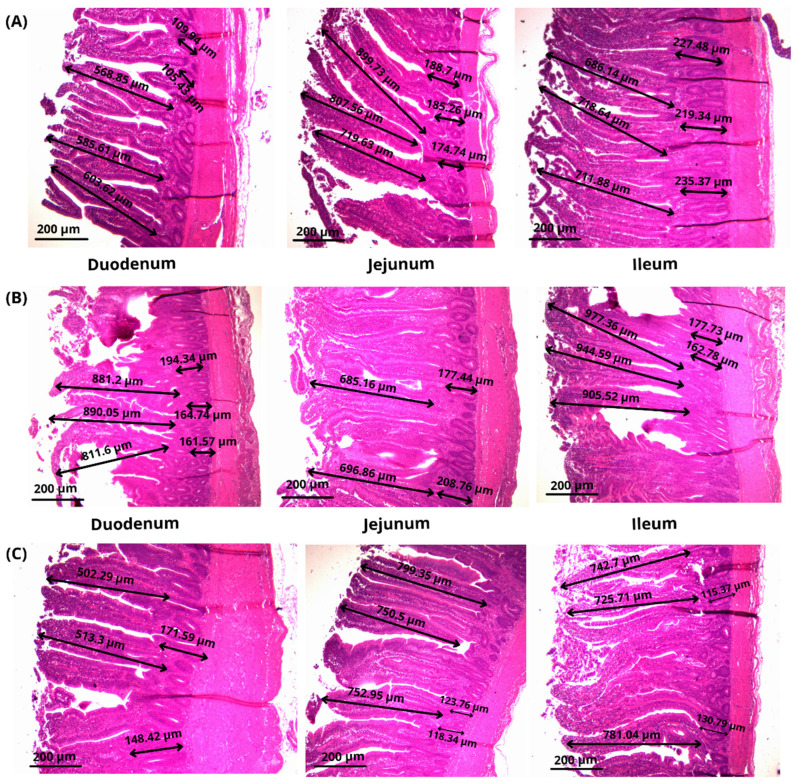
Morphological measurement of villi in Thai native chicken (KKU 1) on duodenum, jejunum, and ileum, fed with citric acid by-product from rice (CABR), (**A**) control, (**B**) CABR 3%, and (**C**) CABR 6%.

Villi histological examinations of the Thai native chicken (KKU 1) are shown in Figure 1 and Table 3. The results showed that the addition of CABR to the diet of Thai broiler chickens (KKU 1) did affect villi histological parameters, including villi height, crypt depth, and villi:crypt ratio (VH, CD, and V:C, respectively). It could be observed that the addition of citric acid by-product (CABR) showed no significant effect in villi height section (duodenum and ileum), but had a significant effect on the jejunum. The addition of 3% and 6% CABR caused a significant decrease effect in CD when compared with the control group (*p* < 0.05), and the addition of CABR has a significant effect on V:C ratio in the duodenum, jejunum, and ileum when compared with the control group (*p* < 0.05).

### 3.3. Microbial Contamination on Fecal Matter

**Figure 2:**
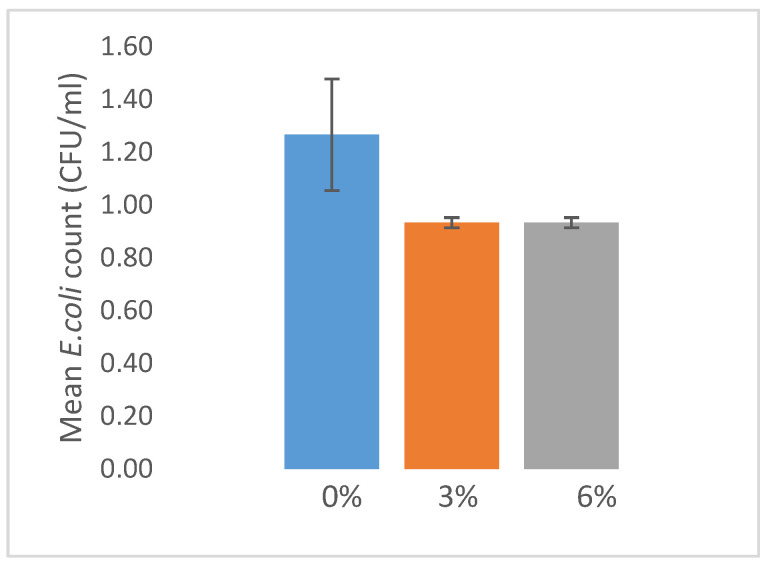
Microbial contamination on fecal matter. Error bars represent ± standard error.

To check for statistical difference of microbial contamination between the treatment, we performed it in the graph (Figure 2). We found that no significant difference between treatment 3% and 6% compared with the control (0%).

## 4. Discussion

The effect of CABR inclusion in Thai native chicken (KKU 1) ration during the starter period (day 1–21 days), grower period (22–49 days), finisher period (50–56 days, and overall period (1–56) days) showed that it did not negatively affect the chicken growth performance, compared with the control group (*p* < 0.05). The result disagreed with the previous research of Tanpong [8], who reported the significant effect of feeding the citric acid by-product from cassava at the level of 3–12% on Japanese quails throughout 1–6 weeks. Citric acid waste from cassava was shown to be a viable replacement at a level of 6% throughout the entire feeding time. However, this study agrees with the result reported by Nourmohammadi et al. [19], who reported that no significant effects on feed intake in broiler chicks fed a diet supplemented with citric acid. FCR result of feeding CABR to KKU 1 chicken showed that it had no significant effect.

Survival rates in this study showed no significant effect on Thai broiler chicken when fed with CABR. Mehdikhany et al. [11] reported that organic acid could lead to a reduction in disease factors in feed, reduction in intestinal pH and harmful microorganisms, and elimination of pathogenic bacteria, and, finally, organic acids appear to cause improvement in the survival rates of animals. Mroz [20], reported that the addition of organic acid to the broiler diet suppressed pathogenic growth and improved digestion, absorption, mucosal immunity, and topical effects on the intestinal brush border. Thompson and Hilton [21] also found organic acid has mainly been used in order to sanitize feed to prevent issues such as salmonella infections in animals. Langhout et al. [22] also found that including organic acids in chicken rations increased performance, such as feed consumption, by strengthening the immune system of the birds, potentially lowering disease risk.

The results of villi histological assessment (Table 3) showed that VH was not affected by the CABR inclusion. Pelicano et al. [23] reported that the increase in the length of the villi is an attempt to increase the surface area of the intestine to maximize absorption once the digestive organs pass through the lumen. However, reduction in villus height, on the other hand, can diminish nutrient absorption by reducing the intestinal surface area available for absorption. As a result, limiting nutrient absorption lowers disease resistance and growth performance [24]. The current finding in this study is in contra with the previous study of Nasibeh et al. [25], who found that supplementation of organic acid has an effect on increasing the villi length of broilers.

CD measurements in this study showed that CABR caused a decrease compared to the control group (Table 3). Deeper crypts indicated tissue turnover in the villi due to normal sloughing, and also to pathogens or toxins/toxin-induced inflammation [26]. Aptekman et al. [27] reported that dietary organic acid were associated with an increase in intestinal nutrient assimilation of broiler diets. Organic acids were shown to enhance the VH in the small intestines and also to have a direct stimulatory effect on the proliferation of gastrointestinal cells. As reported by Tappenden and McBurney, short-chain fatty acids were shown to cause an increase in plasma glucagon-like peptide-2, ileal proglucagon mRNA, and expression of both glucose transporter and celluler protein, all of which are signals that can theoretically mediate the proliferation of gut epithelial cells. These histological improvements in the small intestines possibly led to an increase in the area of the intestinal surface and facilitated the absorption of nutrients to a greater extent, thus enhancing the growth-promoting impact of supplementation with organic acids [28]. Moreover, an increase in the ratio of V:C was detected in this study when compared with the control group (*p* < 0.05). An increase in this ratio could most likely be related to digestion and absorption and cause an increase in the number of beneficial bacteria in the gut lumen [29]. Also, the increase of the ratio V:C might be associated with the increase of the number of beneficial bacteria in the gut lumen [30]. This finding is possibly due to CABR containing the remaining under-graded citric acid which has a low pH and might affect this ratio. This result is critical because a higher ratio of villous height to crypt depth indicates a greater capacity for nutrient digestibility and absorption in chickens. It has been established that shorter intestinal villi relative to crypt depth are associated with a lower number of absorptive cells and a higher number of secretory cells [31].

In the current study the addition of CARB although showing a decreasing tendency of E.coli colony count, no significant differences were observed in comparison to the control treatment. Islam [32] reported that citric acid offers further benefits over antibiotic growth promoters when considering performance, non-specific immunity, and bone formation. Organic acids have antimicrobial effects because they diffuse through the bacterial cell membrane, dissociate into anions and protons, and, eventually, disturb the intracellular electron balance [33]. The incorporation of citric acid, especially at the 4.5 and 6% in drinking water, caused a significant decline of *Bacillus*, *Clostridium*, *Coliform*, and facultative aerobic bacteria, and other bacteria in the gizzard, ceca, and feces [34]. Organic acids reduce the growth of many pathogenic or nonpathogenic bacteria in the gut lumen [35].

## 5. Conclusions

In conclusion, supplementation with CABR produced no negative effects on the growth performance of Thai broiler chickens (KKU 1) and had a significant effect on the villi histological properties of broiler chicks. The addition of CABR did not affect the VH section (duodenum and ileum), but has the effect of decreased CD and increased ratio of V:C when compared with the control group, which indicates that CABR should be considered appropriately when used as a corn replacement in the diets. Moreover, the addition of CABR in feed has no effect in *E.coli* colonies of broiler fecal matter. Therefore, CABR may successfully be used in Thai broiler chicken (KKU 1) diet.

## Figures and Tables

**Table 1:** Feed ingredient of experimental diet period 1–3.

Ingredient (%)	Citric Acid By-Product, % Dry Matter								
	0			3			6		
	Period			Period			Period		
	1	2	3	1	2	3	1	2	3
Corn meal	50.00	55.57	59.47	48.00	53.47	57.37	46.00	51.37	55.27
Soybean meal	26.90	19.30	12.46	25.80	18.30	11.46	24.70	17.30	10.46
Full fat soybean	17.00	19.00	22.00	17.00	19.00	22.00	17.00	19.00	22.00
Dicalcium phosphate % P21	1.80	1.60	1.50	1.80	1.60	1.50	1.80	1.60	1.50
Limestone	1.60	1.40	1.30	1.60	1.40	1.30	1.60	1.40	1.30
DL-Met	0.25	0.20	0.17	0.25	0.20	0.17	0.25	0.20	0.17
Lysine	0.20	0.18	0.15	0.20	0.18	0.15	0.20	0.18	0.15
Rice crude bran oil	1.50	2.00	2.20	1.60	2.10	2.30	1.70	2.20	2.40
Salt	0.30	0.30	0.30	0.30	0.30	0.30	0.30	0.30	0.30
Choline chloride 60%	0.10	0.10	0.10	0.10	0.10	0.1	0.10	0.10	0.10
Premix	0.35	0.35	0.35	0.35	0.35	0.35	0.35	0.35	0.35
Citric acid by-product rice	0.00	0.00	0.00	3.00	3.00	3.00	6.00	6.00	6.00
Total	100.00	100.00	100.00	100.00	100.00	100.00	100.00	100.00	100.00
Calculated									
CP, %	22.39	20.05	18.29	22.33	20.02	18.26	22.26	19.99	18.23
ME, Kcal/kg	3013	3129	3209	3004	3118	3198	2994	3108	3188

**Table 2:** Citric acid by-product from rice (CABR) utilization on growth performance of Thai native chicken (KKU 1) throughout 1–56 days.

Parameter	Citric Acid By-Product Rice, % Dry Matter			*p*-Value
	0	3	6	
Days 1–21				
Initial weight (g/b)	32.77 ± 0.13	32.73 ± 0.22	32.80 ± 0.42	0.9496
Body weight (g/b)	384.16 ± 30.49	392.22 ± 11.60	371.96 ± 26.91	0.5225
BWG (g/b)	351.39 ± 30.59	359.49 ± 11.71	339.16 ± 22.21	0.5249
FI (g/b)	526.36 ± 12.39	514.52 ± 27.59	541.26 ± 30.79	0.3572
FCR	1.51 ± 0.13	1.43 ± 0.07	1.60 ± 0.12	0.1305
SR (%)	100.00 ± 0.00	100.00 ± 0.00	96.88 ± 3.61	0.1004
PI	122.65 ± 18.77	130.72 ± 7.79	107.89 ± 14.82	0.1540
Days 22–49				
Initial weight (g/b)	384.16 ± 30.49	392.22 ± 11.60	371.96 ± 26.91	0.5225
Body weight (g/b)	1461.67 ± 119.71	1475.26 ± 23.39	1423.53 ± 74.98	0.6687
BWG (g/b)	1077.51 ± 100.86	1083.04 ± 20.84	1051.58 ± 52.33	0.7813
FI (g/b)	2171.01 ± 154.87	2158.61 ± 120.24	2091.71 ± 46.42	0.6017
FCR	2.02 ± 0.17	1.99 ± 0.08	1.99 ± 0.13	0.9291
SR (%)	93.75 ± 12.50	95.31 ± 5.98	96.88 ± 6.25	0.8825
PI	140.48 ± 34.44	144.27 ± 11.76	142.14 ± 21.02	0.9759
Days 50–56				
Initial weight (g/b)	1461.67 ± 119.71	1475.26 ± 23.39	1423.53 ± 74.98	0.6687
Body weight (g/b)	1774.09 ± 84.51	1770.13 ± 41.44	1713.49 ± 116.86	0.5631
BWG (g/b)	312.42 ± 46.64	294.88 ± 36.02	289.96 ± 44.29	0.2137
FI (g/b)	875.72 ± 35.63	866.17 ± 57.67	946.18 ± 88.63	0.7649
FCR	2.87 ± 0.53	2.97 ± 0.38	3.30 ± 0.42	0.3240
SR (%)	100.00 ± 0.00	100.00 ± 0.00	100.00 ± 0.00	NA
PI	112.97 ± 19.00	107.92 ± 14.42	94.54 ± 20.16	0.3692
Overall (days 1–56)				
Initial weight (g/b)	32.77 ± 0.13	32.73 ± 0.22	32.80 ± 0.42	0.9496
Body weight (g/b)	1774.09 ± 84.51	1770.13 ± 41.44	1713.49 ± 116.86	0.5631
BWG (g/b)	1741.32 ± 84.64	1737.40 ± 41.58	1680.69 ± 117.07	0.5637
FI (g/b)	3616.28 ± 255.13	3566.13 ± 210.28	3597.24 ± 83.50	0.9364
FCR	2.08 ± 0.18	2.05 ± 0.09	2.15 ± 0.15	0.6254
SR (%)	93.75 ± 12.50	95.31 ± 5.98	93.75 ± 5.10	0.9564
PI	145.08 ± 32.35	147.19 ± 13.96	134.72 ± 21.75	0.7397

BWG = body weight gain; FI = feed intake; FCR = feed conversion ratio; SR = survival rate; PI = production index; and NA = not applicable.

**Table 3:** Morphological measurement of villi in Thai native chicken (KKU 1) on duodenum, jejunum, and ileum, fed with citric acid by-product from rice (CABR).

Parameter	Citric Acid By-Product Rice % Dry Matter			*p*-Value
	0	3	6	
Villi height (µm)				
Duodenum	870.47 ± 81.71	906.87 ± 66.01	913.21 ± 211.12	0.7111
Jejunum	768.45 ± 72.05 ^a^	769.87 ± 70.65 ^a^	659.87 ± 111.45 ^b^	0.0047
Ileum	508.45 ± 60.63	502.48 ± 73.23	482.84 ± 22.94	0.5155
Crypt depth (µm)				
Duodenum	185.59 ± 9.06 ^a^	173.95 ± 18.82 ^a^	158.04 ± 23.91 ^b^	0.0033
Jejunum	257.66 ± 24.73 ^a^	175.70 ± 14.49 ^b^	156.11 ± 28.56 ^c^	0.0001
Ileum	172.00 ± 39.09 ^a^	108.98 ± 20.24 ^c^	142.99 ± 18.02 ^b^	0.0001
Villi: Crypt				
Duodenum	4.68 ± 0.29 ^b^	5.28 ± 0.78 ^ab^	5.81 ± 1.12 ^a^	0.0067
Jejunum	2.99 ± 0.23 ^b^	4.42 ± 0.63 ^a^	4.38 ± 1.26 ^a^	0.0001
Ileum	3.23 ± 1.32 ^b^	4.81 ± 1.30 ^a^	3.41 ± 0.34 ^b^	0.0020

± standard deviation, Figure having different superscripts in the same row differ significantly (*p* < 0.05).

## Data Availability

Data are contained within the article.
